# Cryogenic Super-Resolution Imaging of Local Photocurrent in Photoconductive Infrared Detectors

**DOI:** 10.3390/s26103115

**Published:** 2026-05-15

**Authors:** Lei Ma, Nili Wang, Liaoxin Sun, Dechao Shen, Qianchun Weng, Xiangyang Li, Wei Lu

**Affiliations:** 1 School of Information Science and Technology, ShanghaiTech University, Shanghai 201210, China; malei2023@shanghaitech.edu.cn; 2 School of Materials Science and Technology, ShanghaiTech University, Shanghai 201210, China; 3 National Key Laboratory of Infrared Science and Technology, Shanghai Institute of Technical Physics, Chinese Academy of Sciences, Shanghai 200083, China; wangnili@mail.sitp.ac.cn (N.W.); dcshen@shu.edu.cn (D.S.); qcweng@mail.sitp.ac.cn (Q.W.); lixy@mail.sitp.ac.cn (X.L.); 4 Department of Physics, Shanghai University, Shanghai 200444, China

**Keywords:** HgCdTe, Cryo-SNOM, near-field nano-photocurrent imaging, sub-wavelength defect, local weakening effect

## Abstract

The uniformity of local photoelectric properties in infrared detectors is critical for detection sensitivity. However, micro-nano-scale surface abnormalities introduced during mercury cadmium telluride (HgCdTe) fabrication systematically degrade in-plane photoelectric response consistency. To overcome the optical diffraction limits of standard far-field metrology, we utilized a cryogenic scattering-type scanning near-field optical microscopy (Cryo-SNOM) system to achieve the first super-resolution, in situ imaging of local near-field photocurrent in HgCdTe photoconductive detectors at 10 K. Device-level measurements reveal that sub-wavelength surface protrusions (~tens of nanometers high) act as strong recombination centers, suppressing local photocurrent and causing a consistent 10~20% relative signal attenuation compared to planar regions. Power and bias-dependent testing indicate these defects function as unsaturated linear recombination states. Increasing bias voltage amplifies the coupling between the external field and the defect’s built-in field, broadening the local depletion region and driving a non-linear escalation in the attenuation ratio. This study establishes quantitative engineering tolerances for morphological deviations at the nanoscale, providing critical criteria for the chip integration, structural optimization, and precision manufacturing of high-performance infrared sensing arrays.

## 1. Introduction

Infrared detection technology serves as the fundamental hardware layer for modern information perception and precision control, playing a core strategic role in resource exploration, meteorological monitoring, and national defense security, as well as deep-space astronomical observation, advanced remote sensing, and high-resolution medical thermography [1,2,3]. Among the diverse array of infrared optoelectronic materials, mercury cadmium telluride (Hg_1−x_Cd_x_Te, MCT) has long maintained a dominant position in the engineering of high-performance infrared focal plane arrays (IRFPAs). This is due to its continuous, wide-range tunable bandgap (0.7 eV to 1.6 eV), superior quantum efficiency, and low thermal generation rates [4,5,6,7,8,9,10,11]. However, as optoelectronic systems scale toward ultra-large-scale integration, detector pixel dimensions are rapidly shrinking to sub-wavelength regimes. Within these aggressive scaling parameters, the micro-level uniformity of in-plane local photoelectric properties has emerged as the primary operational bottleneck constraining overall detection sensitivity and array yield.

Despite continuous process optimization in MCT growth, industrial workflows predominantly rely on high-throughput far-field metrology (e.g., macroscopic spectroscopy and macroscopic photocurrent mapping) to benchmark global device quality [12,13]. While these methods effectively evaluate overall performance, the inherent optical diffraction limit obscures a massive density of sub-wavelength surface anomalies within the macroscopic signal footprint. Advanced morphological characterization techniques, such as Scanning Electron Microscopy (SEM) and Atomic Force Microscopy (AFM), have verified that complex fabrication protocols inevitably introduce micro-defects on the MCT surface, including compositional fluctuations, dislocation clusters, and topographical anomalies [10,14]. Yet, constrained by the traditional testing paradigm that decouples morphological inspection from electrical performance evaluation, the quantitative impact of these nanoscale abnormalities on localized electro-optical response remains an empirical blind spot.

Standard localized probing techniques—such as Scanning Photocurrent Microscopy (SPCM), Electron Beam Induced Current (EBIC), and Conductive Atomic Force Microscopy (C-AFM)—face severe hardware limitations when applied to narrow-bandgap infrared detectors operating at cryogenic temperatures. SPCM is strictly bound by diffraction limits, failing to resolve features on the tens-of-nanometers scale. The high-energy electron beams of EBIC induce charge accumulation and severe localized beam damage on the fragile MCT lattice, while hardware integration into 10 K cryogenic environments poses extreme engineering complexities. C-AFM relies on direct physical contact, which easily scores the passivated surface and injects unacceptably high contact leakage currents [15,16,17]. In contrast, scattering-type scanning near-field optical microscopy (s-SNOM) operates in a non-contact tapping mode, eliminating mechanical surface damage while utilizing the localized plasmonic enhancement at the tip apex to achieve super-resolution imaging beyond the diffraction limit. This positions it as the optimal metrology tool for diagnosing nanoscale optoelectronic dynamics in cryogenic operational states [18,19,20]. Compared to the aforementioned techniques, our s-SNOM platform uniquely combines super-resolution, non-destructive tapping, and deep-cryogenic operability. Furthermore, this technique is highly versatile and can be broadly applied to characterize other narrow-bandgap infrared materials (e.g., InSb, Type-II Superlattices), provided their surfaces are sufficiently flat.

Recent s-SNOM applications have successfully mapped plasmon propagation in graphene, carrier scattering in carbon nanotubes, and exciton transport in transition metal dichalcogenides (TMDCs) [21,22,23,24]. While these validations highlight s-SNOM’s utility in evaluating localized charge transport, directly deploying this technique on multi-layered, industrial-grade narrow-bandgap infrared detectors introduces severe device-physics challenges. At room temperature, narrow-bandgap semiconductors generate massive populations of thermal carriers, creating a dark current background that completely eclipses the ultra-weak near-field optoelectronic signal. Therefore, operating at a deep-cryogenic temperature (e.g., 10 K) is strictly necessary to “freeze out” these thermally generated carriers, drastically reduce the dark current, and elevate the signal-to-noise ratio to extract pure photoelectric responses. Consequently, in situ near-field characterization of operational optoelectronic devices at deep-cryogenic temperatures represents a highly critical, yet previously vacant, engineering domain.

This work employs the super-resolution capabilities to comprehensively diagnose the non-uniform response mechanisms of MCT detectors at the sub-wavelength scale. Utilizing a Cryo-SNOM system in a high-vacuum, T = 10 K environment, we report the first synchronous, high-signal-to-noise imaging of local topography and near-field photocurrent distributions across an operational photoconductive MCT chip. By systematically correlating sub-wavelength physical protrusions with localized photocurrent attenuation, we experimentally quantify the localized suppression effects induced by morphological anomalies. Furthermore, we evaluate the specific failure mechanisms and carrier recombination dynamics driven by micro-defects under varying excitation power and bias voltage regimes.

## 2. Materials and Methods

### 2.1. Material Preparation and Device Fabrication

**Figure 1:**
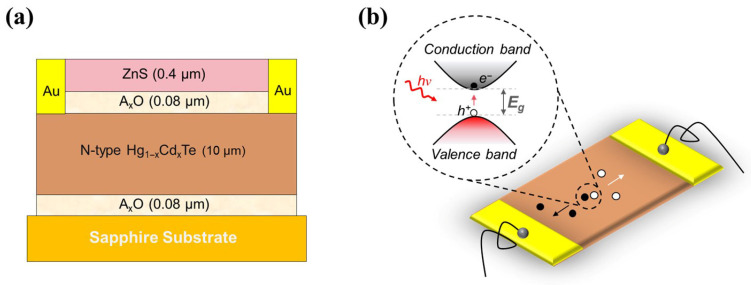
Schematic diagram of the HgCdTe detection chip structure. (**a**) Cross-sectional architecture of the HgCdTe chip; (**b**) Schematic diagram of the physical mechanism of the photoconductive HgCdTe chip.

To enable rigorous near-field electro-optical characterization, targeted fabrication protocols were applied to the MCT infrared detector architecture. Firstly, the MCT materials were synthesized by the solution method, and the bulk single crystals were prepared by the traveling heater method. The wafers subsequently underwent chemical-mechanical polishing, vacuum baking, thermal desorption, and precise dicing to strip mechanical damage layers, residual organics, and native oxides. To satisfy the operational requirements of photoconductive detectors—specifically demanding extended minority carrier lifetimes and high electron mobility—the diced crystals were subjected to extended thermal annealing (~300 °C) in a mercury-rich atmosphere. This process drives Hg atom diffusion into the lattice to annihilate vacancies, transitioning the bulk material to an N-type configuration. Finally, surface oxidation and passivation protocols were executed to critically suppress surface carrier recombination. The complete cross-sectional architecture of the fabricated chip is detailed in Figure 1a.

As shown in Figure 1b, the low-temperature working mechanism of the photonic MCT is as follows: High-energy infrared photons transfer their energy to the electrons in the material. Valence band electrons transition to the conduction band, leaving behind holes. Under the action of an external bias voltage, the electrons and holes move towards opposite electrodes, respectively, thereby forming the internal photoelectric response of the device. Post-fabrication, baseline macroscopic optical and electrical performance metrics of the photoconductive MCT chip were benchmarked. Operating at 253.15 K under a 2 V bias drive, core device parameters were extracted. As summarized in Table 1, the device registers an operating current of 2 mA, a static resistance of 1340  Ω, and maintains an ultra-low noise voltage density of 2.79 × 10^−8^ V/Hz^1/2^. Regarding its infrared optoelectronic response, the device achieves a peak responsivity (R) of 4.33 × 10^3^ V/W and a corresponding peak detectivity ($D^{*}$) of 1.24 × 10^10^ cmHz^1/2^/W. Furthermore, based on normalized photocurrent response measurements, the 50% cutoff wavelength of the MCT detector is located at approximately 5.25 μm at the operating temperature of 253.15 K. According to the classical Hansen-Schmit-Cassel empirical formula, the relationship between the band gap ($E_{g}$), Cd fraction (x), and temperature (T) is defined as [25]:

$$E_{g}\left(x,T\right)=-0.302+1.93x-0.810x^{2}+0.832x^{3}+5.35\times 10^{-4}T\left(1-2x\right)$$

the chemical composition of the grown material is calculated to be Hg_0.732_Cd_0.268_Te. Its corresponding band gap at 253.15 K is 0.236 eV, which narrows significantly to approximately 0.176 eV at the deep-cryogenic operating temperature of 10 K. The incident 6 μm laser (photon energy ~0.206 eV), therefore, strictly provides an above-bandgap excitation at 10 K.

These macroscopic diagnostics verify that the MCT chip exhibits standard infrared photoconductive characteristics and robust photoelectric conversion efficiency, serving as a highly reliable baseline for subsequent nanoscale characterization.

### 2.2. Near-Field Photocurrent Imaging Setup

Nanoscale photoelectric characterization was conducted using a commercial cryogenic scattering-type scanning near-field optical microscopy (Cryo-SNOM) platform (Attocube Systems AG, Haar, Germany).

A mid-infrared quantum cascade laser (QCL) emitting at a wavelength of 6 μm served as the primary optical excitation source. The laser beam was precisely aligned and focused onto the apex of a conductive metallic atomic force microscopy (AFM) probe. The system operated in an amplitude-modulation tapping mode, with the cantilever driven at a mechanical resonance frequency (Ω) of approximately 285 kHz. The metallic tip apex acts as a near-field optical antenna, generating a highly localized and enhanced optical near-field via plasmonic coupling, which consequently excites photocarriers within the sub-wavelength volume of the device lattice directly beneath the tip. The device was driven by an external continuous-wave DC bias voltage applied across the terminal electrodes. The resulting near-field photocurrent was first routed through a low-noise transimpedance amplifier (TIA) for primary current-to-voltage conversion. This raw voltage signal was then further conditioned and amplified by a low-noise voltage preamplifier (SR560, Stanford Research Systems) to optimize the signal strength. Finally, the amplified signal was fed into a lock-in amplifier and demodulated at Ω of the probe’s tapping frequency. Regarding metrological accuracy, the lateral spatial assessment error is ∼±15 nm (governed by the ~20 nm tip radius), while the vertical morphological accuracy is strictly within ±0.5 nm. Optoelectronically, the system’s baseline noise at 10 K introduces a voltage fluctuation of ∼±0.2 mV. The maximum physical limitation of this approach is the finite near-field penetration depth, which restricts the effective probing volume to surface and sub-surface regions (∼50–100 nm deep), rather than deep-level bulk defects.

## 3. Results and Discussion

### 3.1. Synchronous Imaging of Morphology and Photocurrent

**Figure 2:**
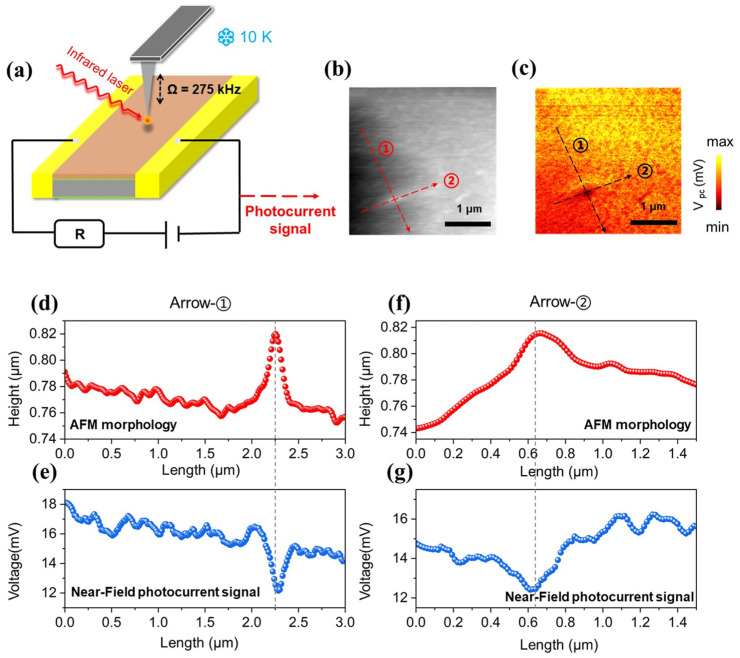
Surface localized near-field photocurrent imaging and morphological correlation analysis of the HgCdTe detection chip. (**a**) Schematic of the near-field photocurrent imaging setup; (**b**) Localized atomic force microscopy (AFM) topography image (Scale bar: 1 μm); (**c**) Synchronous near-field photocurrent image of the corresponding area in (**b**) (Scale bar: 1 μm); (**d**) Morphological height profile along arrow ① in (**b**); (**e**) Near-field photocurrent intensity profile along arrow ① in (**c**); (**f**) Morphological height profile along arrow ② in (**b**); (**g**) Near-field photocurrent intensity profile along arrow ② in (**c**).

To surpass the spatial resolution limits of far-field testing, in situ near-field photocurrent imaging of the MCT chip was executed utilizing an s-SNOM system operating at 10 K in a high-vacuum (<5 × 10^−6^ mbar) chamber. It is necessary to create a highly vacuum environment to prevent the influence of low temperature on the stability of the probe. As illustrated in Figure 2a, a mid-infrared quantum cascade laser emitting at 1601 cm^−1^ was utilized as the excitation source. The infrared beam is locally enhanced by the apex of a metallic probe to excite the sample, generating photocarriers that are subsequently swept and collected by the terminal electrodes. The probe operates in tapping mode at a mechanical frequency of 275 kHz, and high-order harmonic signals are demodulated to strictly filter out far-field background interference, ensuring the synchronous acquisition of high-fidelity topological and near-field photocurrent data. Because both channels are acquired simultaneously by the exact same probe, every pixel of the photocurrent map is strictly spatially locked to the identical coordinate of the physical topography, inherently eliminating any risk of spatial mismatch during interpretation.

To rigorously validate the integrity of the nanoscale photoelectric signal at deep-cryogenic temperatures and eliminate systemic artifacts from far-field stray light, near-field approach curves were extracted prior to large-area scanning. Calibration data demonstrated that as the probe retracts from the device surface, the photocurrent signal undergoes an exponential decay. This standard engineering calibration provides definitive physical evidence that the acquired photocurrent stems entirely from the near-field coupling of localized polaritons at the tip apex, establishing a highly reliable data baseline for evaluating sub-wavelength defect suppression.

As shown in the AFM imaging in Figure 2b, a distinct localized physical protrusion measuring roughly 50 nm in height with sub-wavelength lateral dimensions is clearly identifiable. The synchronously mapped near-field photocurrent image (Figure 2c) reveals a drastic signal degradation at precisely the exact spatial coordinates of the physical protrusion. This empirically demonstrates that structural deformation at the micro-nano scale directly induces severe spatial non-uniformity in the device’s localized photoelectric response.

Line-scan profiles (along arrow ① in Figure 2b,c) further establish the strong electro-mechanical correlation: at the precise peak of the morphological protrusion, the photocurrent registers a distinct local minimum (Figure 2d,e). This indicates that structural undulations exert a profound localized attenuation on photogenerated carriers. This attenuation is fundamentally attributed to a localized aggregation of recombination centers. Cross-directional profiles (along arrow ②, Figure 2f,g) confirm identical behavior. During the fabrication process of MCT detection devices, physical surface protrusions are structurally linked to lattice distortions or localized aggregations of point defects, such as Hg vacancies. Based on Shockley–Read–Hall (SRH) recombination physics [26], these anomalous zones transform into aggressive recombination centers, drastically curtailing local minority carrier lifetimes and forcing massive carrier annihilation prior to electrode collection. Notably, shallow surface roughness (<20 nm) from benign polishing residuals lacks sufficient deep-level defects to disrupt carrier transport. However, protrusions exceeding a critical threshold (~50–60 nm) signify severe lattice distortions that act as dense trap aggregations. This establishes a critical engineering tolerance, demonstrating that only structural defects beyond this morphological threshold will inevitably trigger significant localized signal attenuation.

### 3.2. Impact of Excitation Power on Local Photoelectric Response

**Figure 3:**
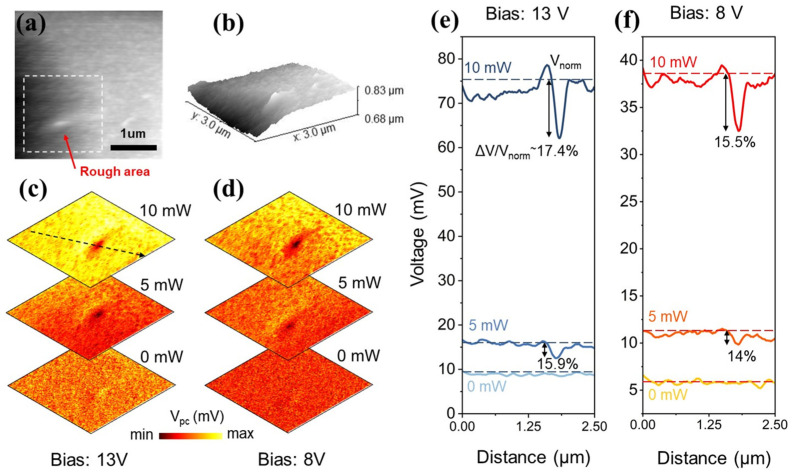
Localized near-field imaging characterization of the HgCdTe detector under varying excitation powers. (**a**) AFM topography image; (**b**) 3D morphological map; (**c**) Near-field photocurrent images at a 13 V bias under different excitation powers (0 mW, 5 mW, 10 mW); (**d**) Near-field photocurrent images at an 8 V bias under different excitation powers (0 mW, 5 mW, 10 mW); (**e**) Near-field photocurrent profile at a 13 V bias (extracted along the dashed black arrow in (**c**,**d**)); (**f**) Near-field photocurrent profile at an 8 V bias.

To systematically investigate how incident excitation power modulates the localized photoelectric response at the nanoscale, variable-power nano-photocurrent mapping (0 mW, 5 mW, 10 mW) was performed on a target region containing a characteristic surface anomaly. Figure 3a details a 3 × 3 μm in-plane AFM scan, where a distinct nanoscale protrusion (indicated by the red arrow) is located within the dashed bounding box. Three-dimensional topographical reconstruction (Figure 3b) specifies the absolute height and spatial geometry of this defect. Under a fixed continuous drive bias (V = 13 V), near-field photocurrent imaging was recorded across varying incident laser powers (Figure 3c).

Extracting the photocurrent profiles along the dashed black arrow in Figure 3c reveals that the global photocurrent scales steadily with increased optical power, strictly adhering to the linear response mechanics of photoconductive devices. However, cross-sectional analysis (Figure 3e) exposes that the localized response at the physical protrusion consistently underperforms compared to the surrounding nominal lattice. Repeating these sub-wavelength power sweeps at a reduced operational bias of 8 V (Figure 3d,f) yielded physically consistent trends.

To engineer a quantitative model of this structure-induced signal degradation, two key diagnostic metrics are defined: Absolute Attenuation (ΔV) and Relative Attenuation Ratio (η):

$$\Delta V=V_{\mathrm{norm}}-V_{\mathrm{rough}}$$

$$\eta =\frac{\Delta V}{V_{\mathrm{norm}}}\times 100\%$$

Here, $V_{\mathrm{norm}}$ is the average photovoltage across the planar regions, and $V_{\mathrm{rough}}$ represents the minimum photovoltage measured at the protrusion. The data indicates that ΔV increases monotonically with rising laser power. From an engineering standpoint, as photon flux increases, this defective zone acts as an operational bottleneck, scaling up total carrier losses proportionally and demonstrating massive absolute suppression under high-intensity illumination. However, analysis of the relative attenuation ratio (η) illuminates the underlying recombination dynamics. Despite multiplier-level increases in optical power, η remains firmly clamped within a 10~20% operational window. This confirms that under current optical injection levels, the defect’s localized recombination centers have not saturated; the capture cross-section remains constant, adhering to a strict linear recombination mechanism. If non-linear optical processes, such as Auger recombination, were dominating the localized carrier dynamics under the intense tip-enhanced field, the recombination rate would scale non-linearly with the carrier density ($R\propto n^{3}$). This would inevitably cause a massive, non-linear distortion of η as the laser power increased. The sustained stability of the η provides solid experimental verification that the primary loss mechanism is the unsaturated SRH recombination rather than nonlinear optical processes. Furthermore, high-bias states (13 V) yield a slightly elevated η (~17.4%) compared to lower bias states (8 V, ~15.5%), indicating that the drive field modulates relative efficiency.

### 3.3. Impact of Bias Voltage on Local Photoelectric Response

**Figure 4:**
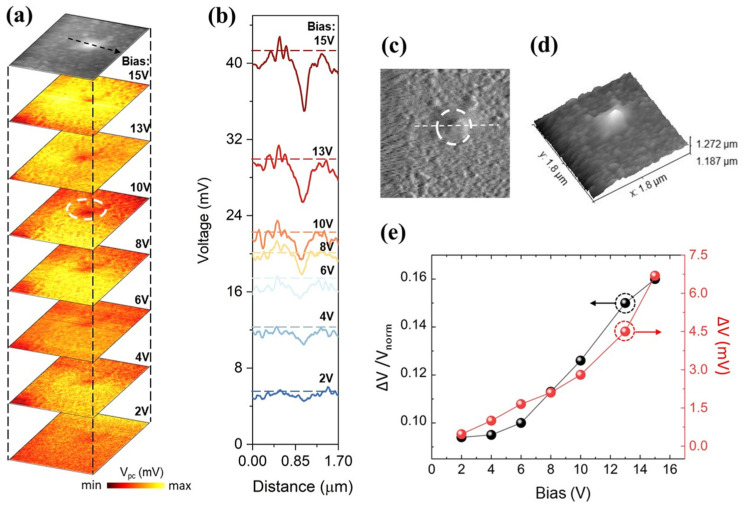
Characterization and analysis of localized near-field imaging of the MCT detector under varying bias voltages. (**a**) Near-field photocurrent images under different bias voltages (2 V, 4 V, 6 V, 8 V, 10 V, 13 V, 15 V); (**b**) Photocurrent intensity profiles along the dashed black arrow in panel a under varying bias voltages, which are continuously tracked at strictly spatially locked coordinates corresponding to the topographical defect; (**c**) Optical contrast image; (**d**) AFM topography image; (**e**) Curves illustrating the relationship of absolute attenuation (ΔV) and relative attenuation ratio (η) as a function of the operating current.

The modulation effects driven by the applied bias voltage require rigorous evaluation. Maintaining a constant excitation threshold (P = 10 mW), near-field photocurrent imaging was conducted across a sweeping bias range from 2 V to 15 V over the target defect region (Figure 4c,d). As illustrated in Figure 4a,b, the total global photocurrent scales quasi-linearly with the applied bias, strictly matching the ohmic contact architecture of the photoconductive device. However, the localized profile analysis (Figure 4e) exposes that the morphology-induced absolute attenuation (ΔV) surges from 0.9 mV at 2 V to approximately 7 mV at 15 V. Critically, the relative attenuation ratio (η) exhibits a non-linear climb, shifting from roughly 9% up to 16% as the bias increases.

When conducting nanoscale near-field photocurrent imaging at deep-cryogenic temperatures, artifacts derived from the photothermal effect (PTE) at the probe apex must be systematically eliminated. Control scans executed at zero bias (V = 0) revealed extremely weak photocurrent signals devoid of any obvious spatial features. If local temperature gradients (PTE) or probe-induced bolometric effects were the driving force, a thermal gradient-induced signal (Seebeck effect) would still persistently manifest at zero bias. The strict requirement of an external bias to extract the spatially dependent signal perfectly aligns with the linear photoconductive mechanism and unequivocally negates thermal generation or tip-heating artifacts. This confirms that the recorded signals are exclusively driven by field-induced photoconductive mechanics. Photoconductive devices generate zero macroscopic current without an external field, while the PTE functions independently of the applied bias, effectively negating PTE as a primary contributor. Furthermore, at the deep-cryogenic threshold of 10 K, the Seebeck coefficient of HgCdTe is exceedingly low; its theoretical thermoelectric potential contribution is far insufficient to interfere with the millivolt-level near-field photoconductive signals.

In N-type doped matrices, surface morphological protrusions are often accompanied by lattice distortions or aggregations of point defects, such as Hg vacancies. These regions act as strong recombination centers, operating as deep-level traps that aggressively capture the majority carriers (electrons). This process repels surrounding electrons and leaves behind ionized donors, carving out a localized depletion region starved of free carriers. This massive charge segregation establishes a highly localized built-in electric field pointing toward the defect core, manifesting as significant localized energy band bending.

When a high external bias voltage is applied, it not only accelerates carrier drift but also violently couples with the localized built-in electric field surrounding the defect. This dynamic coupling effectively widens the physical boundaries of the local depletion region and alters its surrounding localized field distribution. The applied bias forcibly funnels a higher volume of photogenerated carriers into this localized potential well, radically expanding the effective capture cross-section of the micro-defect and thereby exacerbating the Shockley–Read–Hall (SRH) non-radiative recombination process. Consequently, while the global photocurrent baseline scales linearly with voltage, the localized consumption rate of carriers at the defect micro-region scales much faster. Macroscopically, this presents as a non-linear climb in the relative attenuation ratio, although the overall suppression ratio remains strictly bounded within the 10% to 20% threshold.

The phenomenon where the relative attenuation ratio η climbs non-linearly from 9% to 16% with increasing bias can be quantitatively explained from the perspective of micro-depletion region modulation. Upon the application of an external bias $V_{\mathrm{ext}}$, the expansion of the localized depletion width W approximates the following physical relationship: [27]

$$W\approx \sqrt{\frac{2\varepsilon_{s}}{qN_{D}}\left(V_{\mathrm{bi}}+V_{\mathrm{ext}}\right)},$$

where $V_{\mathrm{bi}}$ is the built-in potential and $N_{D}$ is the effective doping concentration. The outward expansion of the depletion width W geometrically inflates the defect’s effective capture radius for photocarriers. Using the standard parameters for our N-type HgCdTe matrix (effective doping concentration $N_{D}=1\times 10^{{16\;\mathrm{cm}}^{-3}}$, and relative permittivity $\varepsilon_{r}=17.5$), quantitative estimation indicates that when the external bias voltage increases from 2 V to 15 V, the local depletion width expands significantly from approximately 622 nm to 1704 nm. This dictates a critical engineering guideline for device architecture: while increasing the operating bias linearly scales the global macroscopic photocurrent, it simultaneously acts as a non-linear multiplier on the spatial response non-uniformity generated by local morphological defects. Therefore, this phenomenon provides a definitive upper limit reference for bias voltage in practical infrared optoelectronic systems when balancing overall responsivity.

## 4. Conclusions

This study reports the first successful experimental realization of nanoscale quantitative imaging of the electro-optical response in photoconductive MCT infrared detectors utilizing cryogenic s-SNOM technology. The hardware diagnostics confirm that sub-wavelength surface roughness structurally forces severe attenuation and localized suppression of the photocurrent. This suppression logic is driven fundamentally by the linear recombination mechanics of the defect center, which is subsequently amplified by the geometric distortion of localized electric fields. This work quantitatively defines the operational thresholds of physical morphology on photoelectric efficiency, supplying vital empirical baseline data and physical models required for the advanced chip integration and precision fabrication of next-generation, high-performance infrared arrays.

## Figures and Tables

**Table 1:** Basic performance parameters of the photoconductive mercury cadmium telluride infrared detector chip (253.15 K).

Noise (V/Hz^1/2^)	Peak Responsivity (V/W)	Peak Detectivity (cmHz^1/2^/W)
2.79 × 10^−8^	4.33 × 10^3^	1.24 × 10^10^

## Data Availability

All the data that supports the plots within this paper and other findings of this study are available from the corresponding author upon request.
